# Caring for the Patient with Severe or Very Severe Myalgic Encephalomyelitis/Chronic Fatigue Syndrome

**DOI:** 10.3390/healthcare9101331

**Published:** 2021-10-06

**Authors:** Jose G. Montoya, Theresa G. Dowell, Amy E. Mooney, Mary E. Dimmock, Lily Chu

**Affiliations:** 1 Dr. Jack S. Remington Laboratory for Specialty Diagnostics, Palo Alto Medical Foundation, Palo Alto, CA 94301, USA; jgmontoya123@gmail.com; 2 Four Peaks Healthcare Associates, Flagstaff, AZ 86004, USA; tdowell@safeaccess.com; 3 Independent Consultant, Riverside, IL 60546, USA; amymooney145@gmail.com; 4 Independent Consultant, Waterford, CT 06385, USA; 5 Independent Consultant, Burlingame, CA 94010, USA; lilyxchu@gmail.com

**Keywords:** myalgic encephalomyelitis/chronic fatigue syndrome (ME/CFS), chronic fatigue syndrome (CFS), myalgic encephalomyelitis (ME), severely affected (housebound) patients, very severely affected (bedbound) patients, chronic disease, primary care, home health care

## Abstract

Myalgic encephalomyelitis/chronic fatigue syndrome (ME/CFS) can cause a wide range of severity and functional impairment, leaving some patients able to work while others are homebound or bedbound. The most severely ill patients may need total care. Yet, patients with severe or very severe ME/CFS struggle to receive appropriate medical care because they cannot travel to doctors’ offices and their doctors lack accurate information about the nature of this disease and how to diagnose and manage it. Recently published clinical guidance provides updated information about ME/CFS but advice on caring for the severely ill is limited. This article is intended to fill that gap. Based on published clinical guidance and clinical experience, we describe the clinical presentation of severe ME/CFS and provide patient-centered recommendations on diagnosis, assessment and approaches to treatment and management. We also provide suggestions to support the busy provider in caring for these patients by leveraging partnerships with the patient, their caregivers, and other providers and by using technology such as telemedicine. Combined with compassion, humility, and respect for the patient’s experience, such approaches can enable the primary care provider and other healthcare professionals to provide the care these patients require and deserve.

## 1. Introduction

Myalgic encephalomyelitis/chronic fatigue syndrome (ME/CFS) is a chronic, multi-system, debilitating disease that affects a million or more Americans of all ages, ethnicities, nationalities, genders, and socioeconomic backgrounds [1]. ME/CFS causes profound fatigue, unrefreshing sleep, cognitive impairment, orthostatic intolerance, pain, sensory sensitivities, gastrointestinal issues, and other bodily symptoms leading to substantial impairment in function. The hallmark symptom is post-exertional malaise (PEM), an exacerbation of symptoms and a further relapse in functioning, following even small physical, cognitive, orthostatic, emotional, or sensory challenges that were previously tolerated. Studies have demonstrated neurological, autonomic, immunological, and energy metabolism dysfunction in ME/CFS [1,2,3].

Prevalence estimates for ME/CFS vary considerably because of factors such as the case definition used, how cases were assessed, and whether the study was community based or not. In their 2020 systematic review and meta-analysis, Lim et al. found an average meta-analysis prevalence of 0.65% in adults with a range of 0.38% to 1.45% [4]. The US prevalence estimate of 1–2.5 M [1] is less than 1% of its population.

Based on studies in the US, UK, and Norway, an estimated 25% of people with ME/CFS are mild and able to work [5] while an estimated 25% are homebound (severe) or bedbound (very severe) [6]. Because they are unable to leave their homes, those who are homebound or bedbound are rarely included in research studies or seen by primary care and other healthcare providers unless there is a crisis, such as a very severe relapse [7] or life-threatening malnutrition [8]. All ME/CFS patients struggle to obtain a correct diagnosis and access the clinical care and support they need because of misunderstanding, a lack of a biomarker, and a lack of proper clinical guidance [1]. As a result, many people with ME/CFS are often not diagnosed or are misdiagnosed. This is especially challenging for severe and very severe patients because they are typically not seen in doctors’ offices. For their part, clinicians may not have seen this level of debility and may not recognize or believe in the disease [8,9,10].

Updated ME/CFS clinical guidance has been published but these have primarily focused on the less severely ill or on children [11,12,13,14,15]. This article combines the published information available on severe and very severe ME in these and other sources [8,10,16,17] with the authors’ experiences with this subgroup of patients and their caretakers. The emphasis on multi-disciplinary care is reflective of the authoring team, which includes two physicians, a health services researcher, an advanced practice nurse, an occupational therapist, and a physical therapist. Our expertise encompasses general medicine, geriatric medicine, public health, and infectious diseases. We have participated in the care of patients severely affected by ME/CFS and other chronic medical conditions in different settings, including in their homes. Four of us also have personal experience as patients or caregivers. 

This article is intended to fill the gap in clinical knowledge and guidance for severe and very severe ME/CFS in adults and reinforce the importance of compassion, humility, and respect in all clinical interactions. Using already existing interventions and resources, primary care providers and other healthcare professionals can meet patients where they are in their sickness and potentially significantly improve their health, quality of life, and function.

## 2. Spectrum of ME/CFS Severity

The severity of specific symptoms and the level of functional impairment seen in ME/CFS can vary widely from person to person and over time. A given patient may experience a combination of symptoms of differing levels of severity-for instance, very severe cognitive and physical impairment coupled with somewhat less severe orthostatic intolerance. A mildly affected patient may be able to work or attend school with accommodations while the most severely affected patient may be bedbound and need total care. This spectrum of severity is described in Table 1:

Recent research studies provide evidence that this classification system is valid and useful [20,21]. Compared to the non-homebound ME/CFS population, homebound patients report more severe and frequent symptoms and greater functional impairment [6]. For instance, compared to less affected patients, the severely ill ambulate fewer steps daily and demonstrate a lower exercise capacity as measured by cardiopulmonary exercise test parameters such as percent peak oxygen consumption [21,22]. The prevalence of psychiatric diagnoses is similar to that observed in other chronic diseases and psychological well-being/functioning may remain relatively intact [23,24,25].

As with ME/CFS in general, recovery is not common [26] and patients with severe and very severe ME/CFS can remain ill for years or decades. Even so, compassionate, high-quality clinical care can help improve the quality of life, decrease the overall symptom burden, and prevent a worsening of the disease.

## 3. Clinical Features Prominent in Severe and Very Severe ME/CFS

The clinical presentation of severe or very severe ME/CFS includes the features seen in those with milder disease, but some features are more prevalent, and all are much more extreme. This includes [8,10,13,14,15,16,17]:

- Profound weakness. May be unable to move or turn over in bed, eat, get to the toilet, etc.
- Reduced or lack of ability to speak or swallow.
- Severe and often almost constant, widespread pain, severe headaches, and hyperesthesia.
- Extreme intolerance to small amounts of physical, mental, emotional, or orthostatic stressors such as sitting, bathing, toileting, eating, speaking. These can trigger post-exertional malaise and increased weakness.
- Hypersensitivity, sometimes extreme, to light, sound, touch, chemicals, or odors. Exposure can increase pain and other symptoms.
- Severe cognitive impairment that may impede the patient’s ability to communicate and understand written materials.
- Severe gastrointestinal disturbances (e.g., nausea, abdominal pain), early satiety, and food intolerances which can impair adequate nutrition.
- Orthostatic intolerance severe enough to prevent upright posture.
- Sleep dysfunction such as unrefreshing sleep, shifted sleep cycles, and fractured sleep.
- Increased prevalence of comorbidities common to ME/CFS (e.g., mast cell activation syndrome, postural orthostatic tachycardia syndrome) and/or complications of being homebound or bedbound (e.g., osteoporosis, constipation, pressure ulcers, aspiration pneumonia, depression, and deconditioning). These can increase disease burden and complicate management.

Compounding the physical debility, patients with severe or very severe ME/CFS are often isolated, sometimes from their own families, and must deal with the complete loss of their former lives and all that defined them [27]. For those who became ill as children or young adults, that is particularly cruel.

## 4. Providing Compassionate Care for Severe and Very Severe ME/CFS

Primary care providers and other healthcare providers may not have seen patients with this level of severity before. The extreme levels of energy limitations, cognitive impairment, pain, and sensory/substance hypersensitivities may be surprising. At the same time, these patients and their caregivers may have been neglected or treated poorly by previous medical providers [1]. As a result, they have had to become their own experts [10]. Recognize and express sympathy for the challenging experiences patients may have faced previously. A patient-centered, collaborative approach to care that is grounded in compassion and respect for the patient in all interactions will be of benefit to everyone [10]. The following approaches can help:

- Plan for the need to see the patient in their home [10,16]. Currently, most severely affected patients live at home. In our experience, home-based care can be more individualized and is preferred by this subgroup and their families.
- Respect the nature and severity of the patient’s disease in all clinical interactions [10]. Ask patients and caregivers beforehand about any factors (e.g., fragrances, fast movements, brightly colored clothes, loud noises, bright lights, and touch) that exacerbate the patient’s specific sensory sensitivities. Minimize these factors as much as possible. Interact with patients at a pace, time of day, and length of time the patient can manage. Even home visits may tax the patient so leverage the caretaker where possible to conserve the patient’s limited energy. Creative approaches may be required if the patient’s ability to speak is limited.
- Accept the validity of the patient’s report of symptoms. Gain the trust of the patient, caregiver, and family. Listen to what they report with understanding and compassion.
- Be honest about the limits of medical knowledge but reassure the patient that you will do what you can to help them.
- Partner closely with the caregiver, if one is involved, and if needed, other healthcare professionals to provide the resources, services, education, and practical help needed by the patient and caregiver. A specialty consultation may help diagnose and manage those aspects of ME/CFS with which you are unfamiliar. Engage a targeted set of other professionals as necessary and as tolerated by the patient. These could include physical therapists, occupational therapists, nurses, home health aides, social workers, and mental health experts. Home visits by optometrists/ophthalmologists and dentists may be required. Ensure these other professionals are knowledgeable about ME/CFS.
- While providing access to essential healthcare providers, care must be taken not to overwhelm the patient with too many providers or too many visits. Where feasible, leverage the caregiver to save the patient’s energy. For example, capitalize on the caregiver’s intimate knowledge of the patient’s needs, preferences, and status. Teach them to provide certain services to minimize the need for additional healthcare providers. Reserve patient visits for those times where patient input is required or there is a need to examine the patient in-person.
- Be alert to caregiver stress. Community resources, local support groups, and respite services for those caring for people with ME/CFS or other chronic diseases may be helpful.
- Some severely ill ME/CFS patients may not have caregivers. Be alert to their non-medical needs, such as their ability to obtain and prepare food.

## 5. Diagnosis and Assessment

- In addition to the diagnostic approaches used for all ME/CFS patients [1,12,13,28,29], the following assessments are particularly important for the person with severe or very severe ME/CFS [8,10,13,14,16,17,30]: Evaluate the patient’s basic and instrumental activities of daily living (ADLs and IADLs) (Table 2). Documenting ADLs has the added benefit of supporting applications for disability.
- Assess the patient’s individual energy limits (their “energy envelope”) [31] and the energy they expend on ADLs and IADLs.
- Investigate medical issues that may be impacting the patient’s symptom burden or level of functioning. These could include over-exertion resulting in PEM, untreated orthostatic intolerance, pain, sleep difficulties, gastrointestinal issues, unrecognized sensory hypersensitivities, recurrent infections, comorbidities, or complications from being homebound or bedbound. Each symptom should be assessed individually to determine whether it is the result of another specific diagnosis that needs to also be treated [8,12,13,16].
- Assess the patient’s psychological status using methods appropriate for chronic disease. Pay attention to affective symptoms (e.g., sadness, worry) and be careful about attributing somatic symptoms (e.g., fatigue, insomnia, gastrointestinal disturbances) to psychological/psychiatric conditions.
- Assess non-medical issues that contribute to the patient’s level of morbidity. Examples include lack of social services, caretaking, transportation, finances, food, and/or supportive devices.

Because ME/CFS is often unrecognized clinically [1], people with severe or very severe ME/CFS have sometimes been stigmatized or misdiagnosed with a mental illness such as anorexia nervosa. Their caregivers have sometimes been accused of neglect or abuse [8,32]. As with other chronic diseases, ME/CFS patients can experience secondary depression and anxiety. They can also be at an increased risk of suicide from the severe functional limitations and severity of symptoms, particularly in the face of medical disbelief and lack of support [33]. However, ME/CFS is not a mental illness [1]. A careful differential diagnosis is required to ensure an accurate diagnosis [13,34]. Concerns for neglect or abuse must be evaluated with full comprehension of the nature of ME/CFS and the level and types of debility that can result. For instance, weight loss or decreased consumption of food and fluids may not be due to intentional self-harm or anorexia nervosa but rather due to undiagnosed gastrointestinal issues that impede nutrition [8].

## 6. Recommendations for Treatment and Management of Severe and Very Severe ME/CFS

Historically, the debility of ME/CFS was incorrectly assumed to be the result of deconditioning that could be treated with graded exercise therapy. However, studies have demonstrated that ME/CFS is not deconditioning [35] and that overexertion can cause harm to patients [36]. This is especially true for people with very severe ME/CFS, for whom even basic ADLs may exceed their extreme energy limits. Thus, recommendations for treatment and management of severe or very severe ME/CFS must be individually tailored to each patient [13,14,15]. These recommendations should be implemented if ME/CFS is suspected, even if the patient has not reached the six-month requirement typical of ME/CFS criteria.

### 6.1. Recommendations for Minimizing Post-Exertional Malaise and Sensory Sensitivities

The following approaches can be used to help manage post-exertional malaise and sensory sensitivities:

- Ensure the patient and caregiver understand post-exertional malaise. Educate them about energy conservation strategies, such as pacing, to minimize the physical, mental, orthostatic, and emotional stressors that could trigger post-exertional malaise with its consequent worsening of symptoms and functioning [11,12,13,37].
- Minimize those stimuli to which the patient is sensitive, such as light, noise, touch, movement, chemicals, and odors, Exposure to these could increase pain and other symptoms (Table 2). The most severe patients may not be able to tolerate any touch, light or noise.
- Accommodate the patient’s restricted energy (Table 2). In the most severe patient, specialized beds, wheelchairs, bedpans, feeding tubes, and catheters may be needed to conserve their extremely limited energy [13,14,16,17].

### 6.2. Recommendations for Treatment and Management Approaches

The following pharmacological and non-pharmacological treatment and management practices can be used to conserve energy, to treat symptoms and comorbidities, and to minimize medical complications [11,12,13,16]. Where possible, leverage the caregiver to minimize the number of providers directly engaging the patient.

- Drugs should be used conservatively and parsimoniously [12,13,16]. When drugs are used, start with very low doses and titrate up slowly as tolerated. For instance, naltrexone is commonly used at 50 mg for opioid overdose but for pain in ME/CFS, the dose starts at 0.1 mg daily and titrates up to 4.5 mg daily. Decrease the risk of side effects and drug–drug interactions by favoring medications which may treat more than one symptom or condition, e.g., both pain and sleep.
- If other pain medications have not been effective or cause significant side effects, it may be necessary to consider opioid medications. Consider starting a medication to counter constipation at the time opioids are prescribed.
- Oral feeding and hydration are preferred and should be tried first. However, tube feeding may be required to ensure nutrition and to conserve the patient’s energy [8]. Intravenous saline may be needed for hydration. If necessary, intravenous feeding may be required as a last resort.
- Physical therapists may help with energy conservation approaches, pain management, joint protection to prevent joint contracture, body positioning, and gentle range of motion, stretching, and strength exercises to help address the effects of being inactive and bedbound (Table 2). The approaches used must be done in such a way that they do not trigger PEM or sensory sensitivities (e.g., to touch) [11,12,13,37]. Caution is advised as even passive straight leg lifts performed by a therapist have been shown to trigger PEM [38]. Caution on stretching is advised for patients with comorbid hypermobile Ehlers-Danlos syndrome.
- Occupational therapists can utilize modification and adaptation strategies for ADLs to conserve energy (Table 2) and to provide patient and caregiver education on techniques for pacing and nonpharmacological approaches to manage symptoms [12,13,37].
- Speech language therapists can help evaluate and treat problems with eating/swallowing as well as problems with communication, whether they stem from anatomical or functional abnormalities in the oral/gastrointestinal tract or in the brain.
- Mental health providers may be able to help patients better cope with the debility of the disease [34].
- Educate the patient, family, and caregiver about helpful behavioral measures. For example, space out caregiving tasks to avoid overstimulation of the patient, adjust/turn the patient occasionally to decrease pressure ulcers, and lower expectations such as the need for a daily bath.

### 6.3. Recommendations for Follow-Up Visits, Advance Care Directives, and Hospitalization

The health status of a severe or very severe ME/CFS patient can change over time, sometimes rapidly and potentially requiring hospitalization. The primary care provider should schedule regular visits, be prepared to provide guidance to hospital staff, and encourage patients to maintain advance directives and contingency plans as follows:

- Schedule follow-up visits on a regular basis. Monitor for emerging comorbidities and complications and whether changes in management practices could help. Do not assume any new issues are caused by ME/CFS or are intractable.
- In the event of a hospital admission, advise staff of the need to provide a low sensory environment and limit the tests and encounters with hospital staff to the extent possible [7,14]. Advise surgeons of necessary precautions for those patients undergoing surgery [39]. Hospitals and clinics may also need information on how to differentiate between ME/CFS and mental illness [13,34].
- Encourage the patient and family to establish a living will, appoint a healthcare proxy, and consider a power of attorney to manage finances if needed. Additionally, encourage them to establish a contingency plan and maintain a summary of their health issues and medications in the event that hospitalization is necessary, or an emergency issue arises. Examples of emergency issues include a fire, loss of a caregiver (e.g., through death or illness), or a very severe relapse in which the patient can no longer communicate their needs.

**Table 2:** Practical Recommendations for Energy Conservation and Management: Summary of ADLs to be evaluated in the bedbound patient and examples of corresponding activity modifications and adaptations that can be employed ^1^.

ADL Domain/Tasks:	Recommendations, Including Modifications/Adaptations ^2^
Grooming/ Washing	Provide shower chair and grab bars. A transfer board can be used to transfer patients from the chair to the tub. Eliminate bathroom mats and rugs that pose a fall risk. Use a tub with a pillow/neck support. Elevate feet and begin with lukewarm water temperatures. Perform sponge bath bedside or in bed to conserve energy. Wash body parts at separate times (e.g., face one day, hair another). Use soaps with low fragrance and that are hypoallergenic. Use dry shampoo. Consider short hair. Examine skin integrity and look for any lesions while bathing. Rest immediately after washing and before dressing if needed. Wrap in blankets, dry towel, or robe and return to bed. Consider bathing every few days instead of daily. Consider remodeling bathrooms to increase accessibility.
Grooming/ Tooth Brushing	Conserve energy by performing activity in bed if needed. Use mild flavor paste or just water. Use a soft-bristle brush. If an electric toothbrush is used, choose one with control for vibration and intensity.
Grooming/ Dressing	Perform activity in bed, if needed to conserve energy. Use fragrance/chemical free laundry detergents. Wear loose fitting clothing made of soft, lightweight, breathable materials. Wear solid colors (no patterns) as these may be less stimulating. Consider adaptive clothing—e.g., slip on, no closures or buttons as these are easier to don (put on)/doff (take off). Don garment on the affected side (e.g., weakest, sorest) first, doff garment on the affected side last. Dress in stages. May not be able to complete all at once. Assess the cause of any sensitivity to clothes—e.g., small fiber neuropathy, contact dermatitis, etc. Change clothes for comfort/cleanliness, not necessarily daily.
Toileting	Use a raised toilet seat and install handrails near the toilet. If needed, a bedside commode can conserve steps for meaningful activity. Use adult diapers, bedpan or catheter when unable to transfer or maintain upright posture. If a catheter is needed, try condom catheters and/or intermittent catheterization first before using long-term in-dwelling catheters. Ask about and plan toileting on a scheduled basis. This can help decrease urgent visits and bladder/bowel accidents.
Feeding and Drinking	Assess whether a patient has food insecurity due to financial, transportation, preparation, or other problems and address as needed. If preparation is the issue, home delivery of meals and/or a supply of frozen or canned foods requiring minimal preparation can be critical, particularly when patients experience bad days. Prepare large quantities of food when able and store for future use. Provide foods that are nutritionally dense and do not need any/much preparation, such as shakes, bars, soft or liquid foods. Referral to a nutritionist may be needed. Provide a variety of snacks that can be easily accessed by the patient. Eat or drink in bed, if needed, to conserve energy. Less severely ill patients may prefer to have a meal(s) with their family for social interaction. Assist with feeding and managing the meal setup if needed. Use lightweight bowls, plates, and utensils (e.g., plasticware, bamboo or other lightweight materials). Use a small, lightweight cup. Use a short straw for less effort to suck. Use a non-spill water bottle or a hydration pack or bag (cut the length of the straw). May require tube feeding for nutrition and hydration or intravenous saline for hydration if oral nutrition and hydration is not adequate.
Positioning and Range of Motion	To protect the patient from pressure sores, joint contractures, skin and joint irritation, and poor alignment: Utilize wedges, bolsters, pillows for support and positioning or consider a specialized/adjustable bed to provide needed support. Switch the head/foot of bed (if needed and possible) to decrease repetitive movements and reaches. Utilize a reclining chair with footrest. Maintain proper neck and lumbar support for proper alignment (e.g., zero gravity chair, lounge chair). Educate caregivers about the need for regular, scheduled re-positioning as tolerated. Utilize passive or active range of motion to help avoid contractures and maintain some flexibility. This must be done in a way that it does not trigger PEM.
Environment/ Room Setup	To protect the patient from undue physical, cognitive, or emotional exertion: Provide a low sensory environment: ○ Hang black-out shades and/or plain curtains (no patterns); ○ Control room temperature and humidity; ○ Limit sounds from inside and outside the home to the extent possible; ○ Do not use products, such as cleaning supplies or perfumes, that have a strong smell. Provide assistive technology such as call buttons; remotes for light, fan and tv control; smart light bulbs (dim/color changing) with remotes; and wireless remote-control electrical outlet switches for fan/lights. Utilize a bedside table with adjustable height, tilt, and swivel top. For ease of reach, use “hook and loop” or similar technology to attach items to the wall and headboard and to position baskets with supplies/snacks/tools within reach. Use magnetic boards, bulletin boards or boards with symbols that people can point to as a communication aid. Assess balance issues, fall risks and hazards (stairs, rugs, home entry, etc.). Remove obstacles to keep pathways open and recommend other mitigation strategies as needed. Provide blankets, fans, and other warming and cooling devices if patients experience poor temperature regulation. If the patient needs to prepare their own meals, organize the kitchen for safety and energy conservation, e.g., provide a stool, position most commonly used dishes and utensils for easy access, etc.
Mobility and Transfers	Provide transfer and mobility devices (e.g., Hoyer lift, slide boards, other assistive devices, wheelchairs, canes, walkers) as required. Use planned, controlled, and slow position changes, especially for people affected by orthostatic intolerance or hypersensitivity to touch. Consider installing a stairlift and/or moving the patient to a more accessible room. Use a wheelchair for transitions between rooms if required and possible. Teach caregivers how to move patients safely. Ask private (e.g., taxi, ride-share) and public (e.g., paratransit, ambulance, fire department) transport services about transport options.
Support and Socialization	Ensure the patient has adequate caregiver support. Help facilitate access to needed community resources. Consider the patient’s desire and need for socialization when recommending energy management approaches.
Medical Management and Emergency Preparedness	Recommend the patient or caregiver create and maintain a summary of their health issues (e.g., symptoms, sensitivities/allergies, cautions for medical services, etc. Tas), current medications (including over-the-counter drugs, supplements, vitamins, etc.), and physician contact information. Recommend advanced directives and a health care proxy for when the patient is unable to convey their intent. Assess emergency preparedness including emergency alert, fire extinguishers, safe exit route. Recommend the patient or caregiver maintain a pack with essential medicine, clothes, and supplies. Recommend emergency alert technology (iWatch, Life Alert, Alexa, etc.) and a cell phone with programmed numbers. Notify emergency services (fire department, police) of resident’s mobility concerns and identify the location as high priority for utility services.

^1^ These recommendations are geared primarily to the bedbound ME/CFS patient but can be tailored as appropriate for patients who are homebound but not bedbound. ^2^ Many of the recommendations address energy conservation and safety issues as these are of particular concern for the severe and very severe ME/CFS patient. For patients who do not have caregivers, the provider will also need to evaluate IADLs such as shopping, cooking, managing medications, and doing laundry and housework to assess the level of support needed [40].

## 7. Practical Considerations for Busy Providers

Caring for such ill patients may be challenging for busy primary care and other healthcare providers. The following approaches can help manage the demands on your time and ensure reimbursement:

- Document ADLs and IADLs to demonstrate the need for home care [40].
- Leverage a combination of home visits, telemedicine, written communications, partnerships with home health care services, partnership with the caregiver if one exists, and emerging remote monitoring technologies to best manage both the needs of the patient and the demands on your time. Delegate tasks which do not need your specific input (e.g., completing repetitive forms, gathering basic information) to clinic staff such as receptionists and medical assistants.
- Be aware of any regulatory or insurance requirements for providing home visits.
- Maximize reimbursement by diagnosing any comorbidities such as postural orthostatic tachycardia syndrome (POTS), Ehlers-Danlos syndrome (EDS), or mast cell activation syndrome (MCAS).

## 8. Pathology in Severe and Very Severe ME/CFS

Research in less severely ill patients has demonstrated dysfunction in neurological, immunological, autonomic, and energy metabolism systems [1,2]. However, few published studies have focused specifically on severe or very severe ME/CFS patients because they are often unable to travel outside their home to participate in research studies. A 2017 review found that only 21 articles had been published on severe or very severe ME/CFS over a span of three decades [41]. Differences in case definition, disease characterization, outcome measures, and small sample sizes made it difficult to draw strong conclusions.

Thus, what is known about severe and very severe ME/CFS has been based largely on clinical experience [8,10,12,13,14,16,17,30] and extrapolated from research utilizing the less severely ill [1,2,3]. However, recent studies in more severe ME/CFS have demonstrated some differences. For instance, compared to mild or moderately ill patients, severely ill patients show lower levels of glycolysis [42] and increased abnormalities in immune markers [43,44]. They also show a reduced number of steps walked per day and lower exercise capacity as measured by percent peak predicted oxygen consumption [22] and a reduction in cerebral blood flow in severe ME/CFS when sitting up at a slight incline [45] or following a 20-degree head-up tilt from a supine position [46].

Future research needs to enable the participation of severe and very severe ME/CFS patients. Methods and tools should be developed to accurately capture the full spectrum of severity. Studies must also assess the pathophysiology, natural history, risk factors and prognosis of severe and very severe ME/CFS. Finally, clinical research should focus on aspects of care unique to or prominent in severe or very severe ME/CFS and how to best address these patients’ needs.

## 9. Conclusions

ME/CFS can cause a wide range of severity and functional impairment with the most severely ill homebound and bedbound, sometimes in need of total care. Yet, as sick as they are, these patients are often not seen by medical providers because they cannot travel to doctors’ offices. Some patients no longer try because they have previously faced disbelief or received treatment recommendations that made them worse. For their part, primary care providers may not have seen this level of debility before. They often lack accurate information on the nature of the disease and how to care for patients with severe or very severe ME/CFS, a problem compounded by the lack of research on these patients.

Caring for such vulnerable patients requires a patient-centered, collaborative approach in all clinical interactions, one that is grounded in compassion, humility, and respect for the nature and severity of the patient’s disease. Use of carefully selected pharmacological and non-pharmacological treatments and management approaches can help protect against worsening of the patient’s health while decreasing symptom burden and improving the patient’s quality of life. Partnerships with the patient, the caregiver and a targeted network of providers along with use of enablers such as telemedicine and remote monitoring are key to providing the needed care without overwhelming either the patient or the busy provider. Using these approaches, the primary care provider can make a significant difference in the lives of these underserved patients. 

## Figures and Tables

**Table 1:** Spectrum of Severity in ME/CFS [12,18,19].

Level of Severity	Description of Level of Functioning and Disease Severity
Mild	Mobile and able to self-care. May be working or attending school, but often with accommodations and by reducing other domestic and social activities.
Moderate	Reduced mobility and restricted activities of daily living. Requires frequent rest periods and typically not working or attending school.
Severe	Mostly homebound. Limited activities of daily living (e.g., self-care, showering, dressing). Severe cognitive difficulties. May be wheelchair dependent.
Very Severe	Bedbound. Unable to carry out most activities of daily living for themselves. Often extreme sensory sensitivity to light, sound, touch, etc. May need total care.

These are general categories intended to convey the wide spectrum of disease severity and functional impairment seen in ME/CFS. The assessment of a given patient should be based on their particular level of disease severity and functional impairment.

## Data Availability

Not applicable.
